# Maternal factors associated with iron deficiency without anaemia in early pregnancy: ECLIPSES study

**DOI:** 10.1007/s00277-023-05123-7

**Published:** 2023-02-15

**Authors:** Lucía Iglesias-Vázquez, Mercedes Gimeno, Pilar Coronel, Ida Henriette Caspersen, Josep Basora, Victoria Arija

**Affiliations:** 1 Nutrition and Mental Health (NUTRISAM) Research Group, Universitat Rovira I Virgili, 43204 Reus, Spain; 2 Institut d’Investigació Sanitaria Pere Virgili (IISPV), 43204 Reus, Spain; 3 Meiji Pharma Spain S.A. (Formerly Tedec-Meiji Farma S.A.), Alcalá de Henares, 28802 Madrid, Spain; 4 Centre for Fertility and Health, Norwegian Institute of Public Health, Oslo, Norway; 5 Tarragona–Reus Research Support Unit, Jordi Gol University Institute for Primary Care Research, 43202 Tarragona, Spain; 6 CIBERobn (Center for Biomedical Research in Physiopathology of Obesity and Nutrition), Instituto de Salud Carlos III, 28029 Madrid, Spain; 7 Collaborative Research Group On Lifestyles, Nutrition, and Smoking (CENIT). Tarragona-Reus Research Support Unit, IDIAP Jordi Gol, 43003 Tarragona, Spain; 8 Tarragona, Spain

**Keywords:** Prenatal, Maternal, Iron status, Iron deficiency, Haemoglobin, Serum ferritin

## Abstract

Several population-specific genetic, sociodemographic, and maternal lifestyle factors are related to iron status in early pregnancy, and their identification would allow preventive actions to be taken. The study aimed to identify maternal factors associated with iron deficiency (ID) in early pregnancy in non-anaemic pregnant women from a European Mediterranean country. Cross-sectional study using the initial population of the ECLIPSES study performed in non-anaemic pregnant women before gestational week 12. Serum ferritin (SF) and haemoglobin concentrations were measured to evaluate iron status, and ID was defined as SF < 15 µg/L. Several sociodemographic and lifestyle data were recorded and used as covariates in the multivariate-adjusted regression models. Out of the 791 participants, 13.9% had ID in early pregnancy. Underweight (OR 3.70, 95%CI 1.22, 15.53) and parity (1 child: OR 2.03, 95%CI 1.06, 3.88; ≥ 2 children: OR 6.96, 95%CI 3.09, 15.69) increased the odds of ID, while a high intake of total meat (≥ 108.57 g/day: OR 0.37, 95%CI 0.15, 0.87), red/processed meat (≥ 74.29 g/day: OR 0.70, 95%CI 0.35, 0.98), protein (≥ 65.05 g/day: OR 0.85, 95%CI 0.30, 0.99), and dietary iron (≥ 8.58 mg/day: OR 0.58, 95%CI 0.35, 0.94) protected against it. Smoking was also associated with a reduction in ID odds (OR 0.34, 95%CI 0.12, 0.99). Baseline BMI, parity, smoking, and diet are associated with ID in early pregnancy in non-anaemic women. Pregnancy planning policies should focus on women at higher risk of ID, such as those who are underweight, multiparous, or following vegetarian diets. This clinical trial was registered at www.clinicaltrialsregister.eu as EudraCT number 2012–005,480-28 and at www.clinicaltrials.gov with identification number NCT03196882.

**Supplementary Information:**

The online version contains supplementary material available at 10.1007/s00277-023-05123-7.

## Introduction

Maternal iron status during pregnancy is a public health concern given the high prevalence of anaemia and iron deficiency (ID) during the gestational period. Estimates indicate that around 25% of pregnant women worldwide suffer from anaemia, mainly caused by ID [1]. Anaemia poses significant problems for maternal and child health [2–4], so haemoglobin levels are routinely monitored to detect it preventively. However, there is a high percentage of pregnant women presenting ID without anaemia who are not diagnosed and monitored in daily clinical practice, as serum ferritin (SF) is not routinely measured [5, 6]. In Europe, 10–33% of pregnant women have ID [7], such as in our study where we have previously observed 14% of participants with ID in early pregnancy [8]. It is therefore of great importance among childbearing women to maintain an adequate iron balance, given the role of iron in multiple physiological processes that take place during pregnancy. Wide evidence supports that prenatal iron imbalances also in absence of anaemia can be detrimental to mother and child. In this regard, previous studies have found that not only anaemia but also ID during the gestational period is associated with preeclampsia, premature births, and even miscarriages [4, 9], as well as physical and cognitive developmental delays in children in postnatal life [3, 10–13].

It is worth mentioning that maternal iron levels in early pregnancy by themselves strongly influence the progression of iron stores during gestation and a woman’s iron status at the end of pregnancy. Indeed, in the ECLIPSES study, we already found a positive association of iron-related biomarker concentrations between the first and third trimester of gestation [8]. Previous studies reached similar findings when assessing the correlation of iron status between early and late pregnancy [14, 15].

Several studies in developed countries have identified some maternal factors that influence the initial iron status of pregnant women, such as sociodemographic, genetic, and lifestyle characteristics [16–19]. However, not all factors that are related have always been analysed together. Many of the risk factors for ID are population-specific, and it is therefore important to disentangle which maternal characteristics place women at risk for ID in each population group. In this regard, few studies have assessed factors associated with iron status in non-anaemic pregnant women.

This study aimed to identify maternal factors associated with ID in early pregnancy in a sample of non-anaemic pregnant women, although with a moderate prevalence of ID, from a European Mediterranean country.

## Material and methods

### Study design and population

The present work included the baseline population of the ECLIPSES study, a community-based randomized controlled trial (RCT) conducted in the province of Tarragona (Catalonia, Spain) [20], before starting the intervention. Briefly, the participants were recruited by midwives in their primary care centres during the routine obstetrical visits prior to gestational week (GW) 12. The main inclusion criteria were over 18 years old and not having anaemia (haemoglobin [Hb] ≥ 110 g/L). Women who had taken > 10 mg iron daily during the 3 months prior to GW12 were excluded.

### Outcome

The study outcome was the iron status of women in early pregnancy and its associated factors. For this, concentrations of iron-related biomarkers (Hb and SF) were measured. Since having anaemia was an exclusion criterion, only ID defined as SF < 15 µg/L according to the WHO guidelines was considered [21].

### Data collection

The research staff recorded the sociodemographic and lifestyle data of the participants during a personal interview using specific questionnaires, including maternal age, baseline body mass index (BMI), smoking habit, ethnicity, parity, pregnancy planning, and use of hormonal contraceptives. The educational level and occupational status of women and their partners were also registered. The family’s socioeconomic status (SES) was calculated from the sociodemographic data of participants and their partners, including educational level and occupational status. Dietary assessment was done using a short food frequency questionnaire (FFQ) validated in our population [22]. Food groups assessed included total meat, red and processed meat, fish, fruits, vegetables, legumes, and dairy products as grams per day (g/day). From this information, energy intake (kcal/day) and nutrients (g/day or mg/day) were calculated using the REGAL (Répertoire Général des Aliments) food composition table [23], complemented by a Spanish food composition table [24]. As for the nutrient intake, protein, fibre, vitamin C, calcium, and dietary iron were assessed. Detailed information is available in Aparicio et al. [25]. Information from the FFQ allowed us to calculate the percentage of adherence to the Mediterranean diet, considered a high–quality dietary pattern [25, 26]. Extended information on data collection can be found elsewhere [8, 20].

Blood samples were taken on GW12 to perform blood and genetic tests. Haematological parameters (Hb and MCV), some specific biochemical markers (SF and C-reactive protein [CRP]), and genetic mutations of the *HFE* gene (C282Y, H63D and S65C) were performed.

### Statistical analyses

Continuous variables (mean and SD) were described using Student’s *t*-test and ANOVA test, while the chi-squared test was used for categorical variables (percentages). Natural logarithm (Ln) transformation was applied to normalize the distribution of SF, increasing the validity of analyses, and using the median and interquartile ranges (IQR). Multivariate regression models (multiple linear regressions and logistic regressions) were used to assess the effect of different prenatal predictors on maternal iron status in early pregnancy. The regression models were adjusted for a wide range of potential confounders, described in the bivariate analyses, including age (< 25 years, 25–35 years, and > 35 years), baseline BMI (underweight, BMI < 18.5; normal weight, BMI 18.5–24.9; overweight, BMI 25–29.9; and obesity, BMI ≥ 30), smoking habit (yes or not), SES (low or middle-high), ethnicity (Caucasian, Latin American, Arab, and Black), parity (primiparous, 1 child, or ≥ 2 children), pregnancy planning (yes or no), use of hormonal contraceptives (yes or no), *HFE* genotype (WT/WT, C282Y/WT, H63D carrier, and S65C carrier), and dietary intake expressed as quartiles. Daily dietary consumption of food groups (total meat, red and processed meat, fish, fruits, vegetables, legumes, and dairy products) and nutrients (protein, fibre, vitamin C, calcium, and iron) were separately included in the regression models to avoid over-adjustment. Daily energy intake as kcal was included in both models. Given that SF can raise in infectious or inflammatory processes, the regression model for SF concentration was additionally adjusted for CRP levels. All statistical analyses were performed using SPSS (version 25.0 for Windows; SPSS Inc., Chicago, IL, USA) and statistical significance was set at *p* < 0.05.

## Results

The study included 791 pregnant women, with a median age of 31 (17–46) years and a median gestational age of 12 weeks at the assessment. Sociodemographic and lifestyle characteristics were presented in Table 1. Regarding the body mass index (BMI), near of 60% of participants had normal weight and more than 40% had excess weight, including overweight and obesity. Most of them were Caucasian (80.4%) and belonged to a middle SES (67%), almost 18% were smokers at the recruitment, and 18.3% reported having used hormonal contraceptives before getting pregnant. For 50.4% of the participants, this was their first pregnancy, and 80% had planned to become pregnant. As for the *HFE* genotype, 33.1% had some mutation, the H63D/WT (26.1%) and C282Y/WT (3.5%) being the most represented genotypes. The less represented genotypes were considered together, leaving the following categories: “WT/WT,” “C282Y/WT,” “H63D carrier” (including C282Y/H63D, H63D/ H63D), and “S65C carrier” (including H63D/S65C, S65C/S65C). In relation to educational level, almost 30% accounted for higher or vocational education, while less than 5% reported having unfinished primary school. Median dietary iron intake was 8 mg/day, and adherence to the Mediterranean diet was high in almost all the participants in the study. A strong statistically significant correlation (Spearman rho 0.915, *p* < 0.001) was found between energy (kcal) and iron intake. Compared to women of normal weight, underweight women reported lower intakes of energy (1827.96 and 1724.57 kcal/day, respectively) and iron (7.97 and 7.40 mg/day, respectively), although the difference was not statistically significant (data not shown).

**Table 1:** Baseline characteristics of the population in the study (*n* = 791)

Age, years	31 [17–46]	Maternal ethnic origin	
< 25	14.7	Caucasian	80.4
25–35	63.3	Latin American	10.7
> 35	22.0	Arab	6.3
Gestational age, weeks	12 [8–12]	Black	2.0
Baseline BMI	25.05 (4.50)	Asian	0.6
Underweight	1.6	Education	
Normal weight	57.8	Unfinished primary school	4.6
Overweight	26.4	Primary school	28.4
Obesity	14.2	Secondary school	38.3
Smoking habit (yes)	17.8	Higher/vocational education	28.7
Use of hormonal contraceptives (yes)	18.3	Familiar SES	
Parity		Low	16.2
Primiparous	50.4	Middle	67.0
1 child	37.6	High	16.8
≥ 2 children	11.9	Food intake	
Pregnancy planning (yes)	80.0	Total meat (g/d)	92 [0–314]
*HFE* genotype		Red and processed meat (g/d)	56 [0–239]
WT/WT	66.9	Fish (g/d)	45 [0–179]
H63D/WT	26.1	Fruits (g/d)	244 [0–929]
C282Y/WT	3.5	Vegetables (g/d)	78 [0–441]
S65C/WT	1.3	Legumes (g/d)	15 [0–60]
C282Y/H63D	1.0	Dairy products (g/d)	294 [0–1530]
H63D/H63D	1.0	Energy (kcal/d)	1788 [853–4290]
H63D/S65C	0.2	Nutrient intake	
S65C/S65C	0.2	Protein (g/d)	56 [10–167]
C282Y/ C282Y	0.0	Fibre (g/d)	13 [3–35]
C282Y/ S65C	0.0	Vitamin C (mg/d)	77 [3–280]
		Calcium (mg/d)	663 [55–2123]
		Iron (mg/d)	8 [2–24]

Data are expressed as mean (SD), median [min–max] and %

*BMI* body mass index, *WT* wild type, *SES* socioeconomic status

Concentrations of SF and Hb, as well as the percentage of women with ID in early pregnancy, were described according to genetic, sociodemographic and lifestyle characteristics (Supplementary Table 1) and by quartiles of dietary intake (Supplementary Table 2). From the overall sample, 13.9% had ID in early pregnancy. Both SF and Hb concentrations increased across increasing BMI categories. In addition, smokers and primiparous women showed higher SF concentrations and a lower percentage of ID than their counterparts early in pregnancy. As for dietary intake, SF concentrations increased progressively across quartiles of total meat and iron intake, whereas a U-shaped distribution was observed for red and processed meat intake, and an inverse U-shaped for calcium intake by quartiles.

Multivariate adjusted analyses showed the effect of prenatal sociodemographic and lifestyle factors on SF and Hb concentrations, and ID at GW12 (Table 2). Parity and being underweight were negatively associated with maternal Hb and SF concentrations, whereas a high intake of total meat (≥ 108.57 g/day), and red and processed meat (≥ 74.29 g/day) increased them in early pregnancy. Additionally, young maternal age (< 25 years) reduced and smoking increased SF concentrations at GW12 but did not show any effect on Hb levels. In relation to ID, smoking, and a high intake of total meat, and red and processed meat, reduced its odds by 66%, 63% and 30%, respectively. Consistent with the observed effect on SF levels, underweight and non-parous women were more likely to suffer from ID in early pregnancy compared to their peers. As for dietary intake, complementarily, when the models were adjusted for daily nutrient intake instead of food groups consumption, a high intake of protein (≥ 65.05 g/day) and iron (≥ 8.58 mg/day) was positively associated with Hb and SF concentrations in early pregnancy, as well as with a reduction in the percentage of ID.

**Table 2:** Associations between early pregnancy concentrations of haemoglobin and serum ferritin and iron deficiency, and predictor variables

	Haemoglobin (g/L)	Serum ferritin (µg/L)	ID (SF < 15 µg/L)
	*β* (95%CI)		OR (95%CI)
Age (years)^a^			
< 25	− 0.28 (− 2.68, 2.11)	− 0.24 (− 0.45, − 0.03)*	1.71 (0.70, 4.18)
25–35	0.00 (Ref.)	0.00 (Ref.)	1.00 (Ref.)
> 35	1.62 (− 0.16, 3.40)	− 0.14 (− 0.30, 0.02)	1.06 (0.56, 2.03)
Smoking			
No	0.00 (Ref.)	0.00 (Ref.)	1.00 (Ref.)
Yes	1.17 (− 0.94, 3.27)	0.08 (0.10, 0.27)*	0.34 (0.12, 0.99)*
Baseline BMI (kg/m^2^)			
< 18.5	− 0.70 (− 2.59, − 0.05)*	− 0.35 (− 0.90, − 0.20)*	3.70 (1.22, 15.53)*
18.5–24.9	0.00 (Ref.)	0.00 (Ref.)	1.00 (Ref.)
25–29.9	0.89 (− 0.92, 2.71)	− 0.04 (− 0.21, 0.12)	0.85 (0.43, 1.67)
≥ 30	1.81 (− 0.44, 4.06)	0.02 (− 0.19, 0.22)	0.80 (0.33, 1.91)
Parity			
Primiparous	0.00 (Ref.)	0.00 (Ref.)	1.00 (Ref.)
1 child	− 2.33 (− 3.99, − 0.68)*	− 0.17 (− 0.31, − 0.02)*	2.03 (1.06, 3.88)*
≥ 2 children	− 2.76 (− 4.78, − 0.46)*	− 0.48 (− 0.71, − 0.25)*	6.96 (3.09, 15.69)*
Total meat intake (g/d)			
< 68.57	0.00 (Ref.)	0.00 (Ref.)	1.00 (Ref.)
68.57–91.73	0.66 (− 1.65, 2.97)	0.12 (− 0.08, 0.31)	0.53 (0.25, 1.12)
91.74–108.56	0.06 (− 2.14, 2.26)	0.12 (− 0.07, 0.30)	0.47 (0.22, 1.02)
≥ 108.57	2.48 (0.25, 4.72)*	0.20 (0.15, 0.33)*	0.37 (0.15, 0.87)*
Red and processed meat (g/d)			
< 37.14	0.00 (Ref.)	0.00 (Ref.)	1.00 (Ref.)
37.14–55.99	0.75 (− 1.62, 3.13)	0.04 (− 0.17, 0.24)	0.42 (0.14, 1.28)
56.00–74.28	0.30 (− 2.62, 2.03)	0.01 (− 0.19, 0.21)	0.55 (0.24, 1.29)
≥ 74.29	1.02 (0.65, 3.91)*	0.16 (0.08, 0.41)*	0.70 (0.35, 0.98)*
Age (years)^b^			
< 25	− 0.28 (− 2.23, 2.78)	− 0.24 (− 0.45, − 0.03)*	1.62 (0.66, 4.01)
25–35	0.00 (Ref.)	0.00 (Ref.)	1.00 (Ref.)
> 35	0.86 (− 1.00, 2.72)	− 0.14 (− 0.30, 0.02)	1.11 (0.57, 2.14)
Smoking			
No	0.00 (Ref.)	0.00 (Ref.)	1.00 (Ref.)
Yes	0.82 (− 1.37, 3.02)	0.08 (0.10, 0.27)*	0.33 (0.11, 0.98)*
Parity			
Primiparous	0.00 (Ref.)	0.00 (Ref.)	1.00 (Ref.)
1 child	− 2.30 (− 4.03, − 0.58)*	− 0.17 (− 0.31, − 0.02)*	2.20 (1.13, 4.28)*
≥ 2 children	− 1.23 (− 3.96, 1.50)	− 0.48 (− 0.71, − 0.25)*	7.39 (3.10, 17.59)*
Protein (g/d)			
< 48.10	0.00 (Ref.)	0.00 (Ref.)	1.00 (Ref.)
48.10–55.92	0.34 (− 0.85, 1.07)	0.12 (− 0.09, 0.33)	0.84 (0.31, 2.27)
55.93–65.04	0.85 (0.52, 2.01)*	0.19 (0.01, 0.37)*	0.70 (0.22, 0.96)*
≥ 65.05	1.06 (0.87, 2.65)*	0.25 (0.10, 0.31)*	0.85 (0.20, 0.99)*
Iron (mg/d)			
< 6.31	0.00 (Ref.)	0.00 (Ref.)	1.00 (Ref.)
6.31–7.67	0.28 (− 0.45, 1.65)	0.06 (− 0.20, 0.15)	0.89 (0.64, 1.98)
7.68–8.57	0.94 (− 0.67, 1,96)	0.26 (− 0.39, 0.42)	0.62 (0.50, 1.32)
≥ 8.58	1.13 (0.21, 2.98)*	0.30 (0.12, 0.73)*	0.58 (0.35, 0.94)*

*ID* iron deficiency, *SF* serum ferritin, *BMI* body mass index

^*^Statistically significant differences compared to the reference group

^a^Adjusted for age (< 25, 25–35, > 35), baseline BMI (< 18.5, 18.5–24.9, 25–29.9, ≥ 30), smoking habit (yes/no), SES (low, middle-high), ethnicity (Caucasian, Latin American, Arab, Black), parity (primiparous, 1 child, ≥ 2 children), pregnancy planning (yes/no), use of hormonal contraceptives (yes/no), *HFE* genotype (WT/WT, C282Y/WT, H63D carrier, S65C carrier), and daily dietary intake (kcal, total meat, red and processed meat, fish, fruits, vegetables, legumes, dairy products)

^b^Adjusted for age (< 25, 25–35, > 35), baseline BMI (< 18.5, 18.5–24.9, 25–29.9, ≥ 30), smoking habit (yes/no), SES (low, middle-high), ethnicity (Caucasian, Latin American, Arab, Black), parity (primiparous, 1 child, ≥ 2 children), pregnancy planning (yes/no), use of hormonal contraceptives (yes/no), *HFE* genotype (WT/WT, C282Y/WT, H63D carrier, S65C carrier), and daily dietary intake (kcal, protein, fibre, vitamin C, calcium, iron)

## Discussion

This study contributes to the identification of some maternal sociodemographic and lifestyle factors associated with the risk of developing ID in early pregnancy in a sample of non-anaemic pregnant women from northeastern Spain, with the aim of preventing this deficiency, which is so common during pregnancy, and to avoid its negative consequences.

From all the analysed maternal biological, sociodemographic, and lifestyle conditions, the most relevant predictor of iron status in early pregnancy identified in this work was multiparity, showing almost 7 times higher odds of ID than primiparous women. That has already been repeatedly reported from around the world [16, 17, 27, 28] and the main explanation is that the high iron cost of pregnancy puts multiparous women at risk of not recovering iron stores from one pregnancy to the next one [29]. Another important predictor of ID in early pregnancy was being underweight, in accordance with previous findings [16, 18, 30, 31]. This association would be indirectly reflecting the effect of poor nutritional status, with low food and nutrient intake. Additionally, underweight women in our study reported lower energy and iron intake than those of normal weight, reinforcing the proposed argument. Other of the studied maternal factors showed a minor impact on the women’s iron status. This is the case of age which, although previous studies have associated younger age with a higher risk of ID [17, 32], being a young mother led to a decrease in SF concentration but did not influence the likelihood of ID in our case. As for the smoking habit, smokers usually show higher levels of SF than non-smokers [19] and, therefore, apparently decrease the odds of ID, as has been found in the present study. According to scientific evidence, cigarette smoking disrupts iron homeostasis inducing a systemic accumulation [19, 33, 34], which leads to the detection of an increase in iron reserves.

This study also assessed dietary intake and its relationship with iron levels. Thus, it was expected that a higher daily intake of total, red and processed meat, as well as protein and iron, would increase both Hb and SF concentrations in early pregnancy, protecting against ID. However, a concern arising from our results is the low iron intake that women reported (median: 8 mg/day), with only 8 participants (1%) meeting the DRI (16 mg/day), and 35.5% not even reaching the EAR (7 mg/day) that EFSA indicates for pregnant women [35]. However, according to a recent review [36], that is not an isolated problem, but the dietary iron intake of most pregnant women in Europe is well below the recommendations. Finally, it has to be stated that, contrary to expected, no effect was found of mutated *HFE* genotypes on iron-related biomarker concentrations or ID in early pregnancy. Despite *HFE* mutations being common in the European population, they are less frequent in Southern Europe [37, 38]. Especially, the variant with high clinical penetrance, *HFE* C282Y homozygous [39, 40], is absent in our study population which may preclude observing some influence of *HFE* mutations on women’s iron status.

A high percentage of women with ID in early pregnancy show no signs of anaemia and their low iron stores go undetected and untreated since Hb is often the only biomarker measured for assessing iron status in routine practice [5, 6]. It is worth mentioning that Hb is the biomarker that is altered the latest when iron status is assessed, as it is only altered when iron stores are already nil and erythropoietic synthesis is unable to synthesise Hb in the required amount. We must therefore consider Hb to be an ineffective biomarker for the prevention of ID during pregnancy, where a decrease in iron levels as pregnancy progresses is the norm unless preventive iron supplementation is carried out. Therefore, some women, even if they have normal Hb levels, may have a high chance of developing ID and anaemia later in pregnancy, with negative consequences for their health and that of their baby. Otherwise, measurement of SF concentrations, which is not common in clinical practice, would provide valuable information on iron stores, including incipient iron deficiency states. The concentration of SF is internationally recognized as a very robust biomarker for assessing iron reserves; when low, there is no possibility of false positives for ID and, although it is true that it may increase in presence of infectious/inflammatory processes, the analyses in our study were adjusted for CRP concentration to account for acute infections.

It must be said that deciphering which factors are associated with ID in early pregnancy does not mean that iron stores should not continue to be monitored throughout pregnancy. However, it has been observed that early iron status is highly correlated with iron status during and at the end of gestation. Therefore, it is in the early stages of pregnancy, and even periconceptionally, that effective preventive actions can be considered, such as increased promotion of healthier lifestyles from pregnancy planning services and increased monitoring of non-modifiable characteristics where appropriate. Thus, knowing which maternal conditions or characteristics may affect maternal iron stores would allow obstetricians and midwives to focus public health actions on the target population for early prevention of ID, helping women to achieve the best possible iron status.

The strengths of the present work include (1) the extensive data collection carried out, which covered many sociodemographic and lifestyle characteristics of the participants, including diet and toxic habits; (2) SF levels were adjusted for CRP concentrations in multivariate analyses allowing control for acute inflammatory processes at the time of measurements that could bias the results. However, some limitations must be considered when interpreting these findings. First, the observational design of the study may influence the external validity of the results. On the assumption that the sample includes only non-anaemic women in early pregnancy, the current findings cannot be extrapolated to other populations. Second, information on recent blood donations, which would reduce iron reserves, was not available. Third, dietary assessment using questionnaires is susceptible to misreporting bias; however, given that women with low BMI in our study did not over-report food intake, as is often the case, we believe that potential misreporting bias would not greatly influence our results. Finally, information about interpregnancy interval was not available, which could have allowed further interpretation of parity as a predictor of iron status.

## Conclusion

Since Hb concentration is very often the only biomarker of iron status used in clinical practice, pregnant women with anaemia are treated with iron supplementation, but those with ID without anaemia remain under-diagnosed and untreated, so estimating SF concentration in early pregnancy and its associated factors is of great importance for early prevention of ID. Multiparity and being underweight are strong predisposing factors of maternal ID in early pregnancy in non-anaemic women. A diet high in meat, protein, and iron reduce the likelihood of starting pregnancy with ID. Midwives and obstetricians should pay special attention to the iron status of pregnant women at high risk of ID, such as those who are underweight, multiparous or on vegetarian diets.

## Supplementary Information

Below is the link to the electronic supplementary material.

Supplementary file1 (DOCX 38 kb)

## Data Availability

The datasets analysed during the current study are available from the corresponding author on reasonable request.
