# Supplementary material for: Maternal factors associated with iron deficiency without anaemia in early pregnancy: ECLIPSES study
Source: Ann Hematol. 2023 Feb 15;102(4):741–8. doi: 10.1007/s00277-023-05123-7 (PMC9998312; doi:10.1007/s00277-023-05123-7)
Supplement: Supplementary file 1 — Supplementary file1 (DOCX 38 kb) [file 277_2023_5123_MOESM1_ESM.docx]

**Supplementary Table 1. Baseline serum ferritin and haemoglobin concentrations and percentage of iron deficiency by sociodemographic and lifestyle factors**

|  |  | **Haemoglobin**  **(g/L)** | |  | **Serum ferritin**  **(µg/L)** | |  | **ID (SF<15 µg/L)** |  |
| --- | --- | --- | --- | --- | --- | --- | --- | --- | --- |
|  |  | Mean (SD) | Median [IQR] |  | Mean (SD) | Median [IQR] |  | % |  |
| All |  | 127.59 (7.76) | 128 [122-132] |  | 41.82 (29.99) | 39 [21-49] |  | 13.9 | |
| Age (years) |  |  |  |  |  |  |  |  | |
| <25 |  | 128.28 (7.87) | 129 [122-129] |  | 38.75 (30.62) | 38 [19-44] |  | 16.4 | |
| 25-35 |  | 127.57 (7.74) | 127 [122-132] |  | 43.67 (29.52) | 42 [23-54] |  | 12.8 | |
| >35 |  | 127.57 (7.74) | 128 [122-132] |  | 38.56 (30.64) | 33 [20-43] |  | 15.5 | |
| Education |  |  |  |  |  |  |  |  | |
| Unfinished primary school |  | 126.00 (8.26) | 126 [122-130] |  | 44.59 (37.70) | 36 [18-52] |  | 16.7 | |
| Primary school |  | 127.56 (7.72) | 128 [122-132] |  | 40.35 (28.68) | 38 [20-47] |  | 15.6 | |
| Secondary school |  | 127.35 (7.61) | 128 [122-133] |  | 43.49 (31.28) | 42 [23-52] |  | 11.6 | |
| Higher/vocational education |  | 128.19 (7.90) | 128 [123-132] |  | 40.62 (28.17) | 38 [21-50] |  | 15.0 | |
| Familiar SES |  |  |  |  |  |  |  |  | |
| High |  | 128.04 (7.66) | 128 [123-132] |  | 43.11 (31.31) | 42 [24-45] |  | 13.5 | |
| Middle |  | 127.57 (7.80) | 127 [122-133] |  | 41.70 (29.19) | 40 [21-50] |  | 13.4 | |
| Low |  | 127.21 (7.69) | 128 [123-132] |  | 40.98 (32.04) | 34 [18-45] |  | 16.4 | |
| Ethnicity |  |  |  |  |  |  |  |  | |
| Caucasian |  | 127.83 (7.80) | 127 [122-133] |  | 40.82 (25.80) | 39 [23-50] |  | 12.1 | |
| Latin American |  | 127.17 (7.56) | 127 [122-133] |  | 41.92 (31.95) | 35 [19-45] |  | 17.0 | |
| Arab |  | 127.39 (8.83) | 129 [120-133] |  | 34.74 (24.99) | 32 [15-42] |  | 22.6 | |
| Black |  | 120.00 (7.47) | 118 [115-127] |  | 41.42 (24.48) | 36 [22-63] |  | 20.0 | |
| *HFE* genotype |  |  |  |  |  |  |  |  | |
| WT/WT |  | 127.65 (7.60) | 127 [123-132] |  | 41.49 (32.04) | 34 [20-50] |  | 15.2 | |
| C282Y/WT |  | 126.18 (6.19) | 127 [121-131] |  | 43.62 (35.29) | 31 [15-68] |  | 22.7 | |
| 63D carrier |  | 127.19 (8.13) | 127 [121-133] |  | 42.05 (30.43) | 36 [21-55] |  | 14.2 | |
| S65C carrier |  | 127.40 (12.55) | 126 [117-138] |  | 41.42 (28.63) | 35 [22-55] |  | 20.0 | |
| Baseline BMI (kg/m^2^) |  |  |  |  |  |  |  |  | |
| <18.5 |  | 123.23 (6.80)*^†‡^ | 125 [118-130]*^†‡^ |  | 35.86 (14.87)*^†‡^ | 38 [19-44]*^†‡^ |  | 15.4*^†‡^ | |
| 18.5-24.9 |  | 127.12 (7.67)* | 127 [122-132]* |  | 41.13 (27.99)* | 39 [21-49]* |  | 14.0* | |
| 25-29.9 |  | 127.62 (7.63)^†^ | 128 [122-133]^†^ |  | 42.34 (30.54)^†^ | 39 [21-53]^†^ |  | 14.4^†^ | |
| ≥30 |  | 129.29 (8.19)^‡^ | 129 [123-135]^‡^ |  | 43.48 (36.94)^‡^ | 41 [25-57]^‡^ |  | 12.5^‡^ | |
| Smoking |  |  |  |  |  |  |  |  | |
| Yes |  | 128.39 (7.64) | 127 [123-134] |  | 47.95 (32.08)* | 42 [27-57]* |  | 6.4* | |
| No |  | 127.41 (7.78) | 128 [122-132] |  | 40.49 (29.38)* | 37 [20-46]* |  | 15.5* | |
| Parity |  |  |  |  |  |  |  |  | |
| Primiparous |  | 128.08 (7.95) | 128 [122-133] |  | 45.05 (30.91)*^†^ | 42 [24-54]*^†^ |  | 9.1*^†^ | |
| 1 child |  | 126.91 (7.64) | 127 [122-132] |  | 39.49 (27.44)* | 34 [20-47]* |  | 15.6* | |
| ≥2 children |  | 127.16 (7.18) | 127 [122-132] |  | 34.05 (32.15)^†^ | 28 [14-42]^†^ |  | 30.1^†^ | |
| Pregnancy planning |  |  |  |  |  |  |  |  | |
| Yes |  | 127.79 (7.60) | 128 [123-133] |  | 42.48 (30.77) | 40 [21-50] |  | 13.3 | |
| No |  | 126.77 (8.34) | 127 [121-132] |  | 39.19 (26.59) | 37 [21-45] |  | 16.5 | |
| Hormonal contraceptive use |  |  |  |  |  |  |  |  | |
| Yes |  | 128.22 (7.95) | 128 [123-134] |  | 41.18 (29.59) | 36 [20-50] |  | 16.0 | |
| No |  | 127.40 (7.67) | 128 [122-132] |  | 41.96 (30.15) | 39 [22-49] |  | 13.5 | |

ID, iron deficiency; SF, serum ferritin; SES, socioeconomic status; BMI, body mass index

Statistically significant differences between groups are indicated with the superscript symbols *, †, and ‡.

**Supplementary Table 2. Baseline serum ferritin and haemoglobin concentrations and percentage of iron deficiency by dietary intake**

|  |  | **Haemoglobin**  **(g/L)** | |  | **Serum ferritin**  **(µg/L)** | |  | **ID (SF<15 µg/L)** |
| --- | --- | --- | --- | --- | --- | --- | --- | --- |
|  |  | Mean (SD) | Median [IQR] |  | Mean (SD) | Median [IQR] |  | % |
| **Food intake** |  |  |  |  |  |  |  |  |
| Total meat (g/d) |  |  |  |  |  |  |  |  |
| <68.57 |  | 126.26 (8.57) | 126 [123-133] |  | 38.77 (24.05)* | 38 [21-46]* |  | 15.9 |
| 68.57-91.73 |  | 127.20 (7.35) | 127 [122-132] |  | 42.22 (29.71) | 38 [20-50] |  | 12.7 |
| 91.74-108.56 |  | 127.41 (7.57) | 127 [122-132] |  | 44.21 (34.78) | 39 [22-52] |  | 13.8 |
| ≥108.57 |  | 128.47 (8.31) | 128 [122-132] |  | 45.01 (30.51)* | 42 [24-57]* |  | 13.2 |
| Red and processed meat (g/d) |  |  |  |  |  |  |  |  |
| <37.14 |  | 126.60 (8.36) | 126 [121-132] |  | 41.87 (31.56)* | 38 [19-50]* |  | 18.4 |
| 37.14-55.99 |  | 127.97 (7.50) | 128 [123-133] |  | 42.09 (29.52) | 38 [20-51] |  | 11.9 |
| 56.00-74.28 |  | 127.33 (8.18) | 127 [122-133] |  | 43.21 (28.06) | 39 [24-46] |  | 11.8 |
| ≥74.29 |  | 127.51 (8.44) | 128 [121-132] |  | 45.36 (32.83)* | 42 [23-57]* |  | 13.6 |
| Fish (g/d) |  |  |  |  |  |  |  |  |
| <28.57 |  | 128.01 (7.67) | 128 [123-133] |  | 39.51 (25.44) | 39 [21-46] |  | 16.7 |
| 28.57-45.30 |  | 127.84 (7.41) | 128 [123-132] |  | 44.43 (33.61) | 42 [22-50] |  | 10.6 |
| 45.31-57.13 |  | 127.87 (8.26) | 128 [122-133] |  | 43.00 (32.38) | 39 [22-52] |  | 13.0 |
| ≥57.14 |  | 126.75 (7.65) | 127 [122-132] |  | 40.55 (28.10) | 36 [21-49] |  | 15.2 |
| Fruits (g/d) |  |  |  |  |  |  |  |  |
| <142.85 |  | 127.84 (7.42) | 127 [123-132] |  | 40.57 (28.63) | 37 [21-43] |  | 14.0 |
| 142.85-243.59 |  | 127.64 (8.65) | 127 [121-133] |  | 43.15 (32.00) | 37 [20-54] |  | 17.1 |
| 243.60-314.28 |  | 127.08 (7.40) | 127 [122-132] |  | 44.79 (34.29) | 42 [23-52] |  | 11.5 |
| ≥314.29 |  | 127.89 (7.59) | 128 [123-132] |  | 38.34 (23.04) | 40 [21-45] |  | 13.5 |
| Vegetables (g/d) |  |  |  |  |  |  |  |  |
| <51.43 |  | 128.26 (7.80) | 129 [123-133] |  | 38.96 (28.38) | 36 [19-43] |  | 18.1 |
| 51.43-77.93 |  | 126.89 (7.54) | 127 [122-132] |  | 43.15 (28.07) | 40 [24-43] |  | 9.9 |
| 77.94-91.42 |  | 127.83 (7.50) | 127 [122-132] |  | 42.84 (33.80) | 38 [22-47] |  | 12.8 |
| ≥91.43 |  | 127.59 (7.76) | 128 [122-133] |  | 42.27 (29.23) | 42 [20-54] |  | 14.8 |
| Legumes (g/d) |  |  |  |  |  |  |  |  |
| <8.57 |  | 126.46 (8.64) | 127 [120-131] |  | 39.32 (25.59) | 37 [24-44] |  | 11.9 |
| 8.57-15.30 |  | 128.71 (7.46) | 129 [124-133] |  | 41.35 (27.63) | 39 [22-49] |  | 12.6 |
| 15.31-17.14 |  | 127.25 (7.60) | 127 [122-133] |  | 45.80 (36.99) | 42 [22-54] |  | 12.6 |
| ≥17.15 |  | 127.37 (7.64) | 127 [122-132] |  | 41.44 (29.81) | 40 [20-53] |  | 15.8 |
| Dairy products (g/d) |  |  |  |  |  |  |  |  |
| <230 |  | 126.83 (8.03) | 127 [20-49] |  | 41.95 (29.03) | 38 [22-50] |  | 15.0 |
| 230-293.69 |  | 126.95 (7.89) | 127 [121-132] |  | 41.26 (28.91) | 40 [20-49] |  | 14.0 |
| 293.70-354.99 |  | 127.95 (7.62) | 128 [123-133] |  | 43.52 (32.56) | 42 [22-52] |  | 12.8 |
| ≥355 |  | 128.38 (7.48) | 128 [123-134] |  | 40.06 (28.55) | 37 [21-45] |  | 14.1 |
| **Energy (Kcal/d)** |  |  |  |  |  |  |  |  |
| <1572.94 |  | 127.69 (7.49) | 127 [121-132] |  | 40.32 (29.33) | 38 [20-49] |  | 16.2 |
| 1572.94-1787.99 |  | 127.13 (8.30) | 126 [121-132] |  | 41.98 (30.22) | 36 [25-47] |  | 12.1 |
| 1788.00-1943.50 |  | 127.87 (7.76) | 128 [122-133] |  | 43.54 (32.45) | 42 [23-52] |  | 12.1 |
| ≥1943.51 |  | 127.57 (7.55) | 128 [122-132] |  | 41.24 (27.61) | 42 [21-56] |  | 15.2 |
| **Nutrient intake** |  |  |  |  |  |  |  |  |
| Protein (g/d) |  |  |  |  |  |  |  |  |
| <48.10 |  | 125.34 (8.00)*^†^ | 125 [120-131]*^†^ |  | 39.05 (25.37)*^†^ | 37 [21-45]*^†^ |  | 15.8*^†^ |
| 48.10-55.92 |  | 126.36 (7.50) | 126 [120-130] |  | 42.29 (34.05) | 38 [21-53] |  | 12.5 |
| 55.93-65.04 |  | 127.66 (8.11)* | 127 [122-133]* |  | 44.40 (33.31)* | 42 [24-51]* |  | 11.1* |
| ≥65.05 |  | 128.19 (8.57)^†^ | 128 [122-133]^†^ |  | 45.96 (28.53)^†^ | 42 [21-56]^†^ |  | 10.8^†^ |
| Fibre (g/d) |  |  |  |  |  |  |  |  |
| <10.25 |  | 127.61 (7.60) | 127 [122-132] |  | 40.70 (28.31) | 38 [21-49] |  | 14.3 |
| 10.25-12.82 |  | 127.75 (8.69) | 127 [121-134] |  | 39.20 (27.22) | 34 [21-49] |  | 15.3 |
| 12.83-14.71 |  | 127.71 (7.69) | 127 [122-132] |  | 45.77 (35.97) | 42 [23-54] |  | 11.7 |
| ≥14.72 |  | 127.29 (7.24) | 127 [121-132] |  | 40.26 (25.16) | 42 [22-55] |  | 15.1 |
| Vitamin C (mg/d) |  |  |  |  |  |  |  |  |
| <53.82 |  | 127.87 (7.71) | 127 [122-132] |  | 42.34 (31.09) | 37 [21-52] |  | 13.7 |
| 53.82-77.31 |  | 127.58 (7.79) | 127 [121-133] |  | 38.81 (24.41) | 36 [23-48] |  | 14.5 |
| 77.32-94.07 |  | 127.44 (7.39) | 127 [121-132] |  | 45.13 (33.77) | 42 [23-55] |  | 12.1 |
| ≥94.08 |  | 127.59 (7.76) | 127 [122-132] |  | 40.22 (28.60) | 42 [21-54] |  | 15.7 |
| Calcium (mg/d) |  |  |  |  |  |  |  |  |
| <531.52 |  | 126.81 (7.66) | 126 [121-131] |  | 39.68 (27.60) | 37 [21-49] |  | 16.2 |
| 531.52-662.65 |  | 127.64 (8.22) | 127 [121-132] |  | 45.04 (33.10)* | 42 [23-55]* |  | 12.8 |
| 662.66-760.35 |  | 127.96 (7.31) | 128 [122-133] |  | 44.31 (32.27)^†^ | 42 [25-55]^†^ |  | 10.4 |
| ≥760.36 |  | 127.89 (7.96) | 128 [122-133] |  | 38.44 (26.34)*^†^ | 34 [19-52]*^†^ |  | 16.5 |
| Iron (mg/d) |  |  |  |  |  |  |  |  |
| <6.31 |  | 128.22 (7.81) | 127 [122-133] |  | 40.03 (29.26)* | 37 [20-46]* |  | 17.3 |
| 6.31-7.67 |  | 127.49 (7.96) | 128 [121-132] |  | 40.79 (26.83)^†^ | 37 [25-48]^†^ |  | 11.1 |
| 7.68-8.57 |  | 127.32 (7.61) | 127 [122-132] |  | 46.71 (36.16)*^†^ | 42 [23-54]*^†^ |  | 11.2 |
| ≥8.58 |  | 127.36 (7.72) | 127 [121-133] |  | 45.30 (34.66) | 39 [21-55] |  | 15.9 |

ID, iron deficiency; SF, serum ferritin

Statistically significant differences between groups are indicated with the superscript symbols * and †.
